# Gold hard anodized (GHA) materials with antimicrobial surface properties: mechanical, tribological, and microbiological characterization

**DOI:** 10.1007/s42247-021-00180-y

**Published:** 2021-02-09

**Authors:** Anna Nastruzzi, Franco Cicerchia, Annalisa Fortini, Claudio Nastruzzi

**Affiliations:** 1 G. H. A. Europe s. r. l., Zola Predosa, Bologna, Italy; 2 Department of Engineering, University of Ferrara, via Giuseppe Saragat 1, 44122 Ferrara, Italy; 3 Department of Chemical and Pharmaceutical Sciences, University of Ferrara, via Luigi Borsari 46, 44121 Ferrara, Italy

## Abstract

Infections acquired in public spaces (i.e., transports, restaurants, and bars, hospitals) present a serious burden for the entire health systems. In this respect, appropriate preventative and control measures in order to eliminate or reduce the negative effects of surface-transmitted infections appear highly desirable. Alongside recommendations for treatment and hygiene, antimicrobial material surfaces can offer indeed an important contribution to the prevention of infections. The aim of the current paper is therefore to describe the preparation and characterization of a new material obtained by an innovative anodic oxidation, defined as golden hard anodizing GHA. The anodic oxide surface thanks to the nanoporous structure acts as reservoir of silver ions (Ag^+^) which in turn confer antimicrobial properties to the material surface. Specifically, the manuscript presents a thorough preparation and characterization of a new material obtained by an innovative anodic oxidation treatment applied on commercially available aluminum alloys including the microscopic analysis and the description of the antimicrobial performances against a number of microorganisms, including among the others, Gram-positive (*Staphylococcus aureus*) and Gram-negative (*Escherichia coli*) bacteria. More specifically, the current article describes some of the properties of GHA materials. The tribological properties of GHA were evaluated through experimental tests performed with a pin-on-disk tribometer. The morphology of the wear surfaces was studied by means of a scanning electron microscope (SEM) analysis and profilometry investigations. Furthermore, in order to evaluate the possible anticorrosive properties of GHA, tests in neutral salt spray are in addition described.

## Introduction

The current standard methods of disinfection for surfaces (especially those present in hospitals, schools and public transport vehicles) appear to be largely insufficient. In this respect, a recently published review revealed that very few guidelines or legislation exist for infection propagation control in public spaces [1]. It should be indeed considered that contamination can occur either by direct contact with an infected patient, but also indirectly through a contaminated object or surface [2]. In this respect, surfaces represent an important way of transmission of diseases since they can act as a reservoir of microorganisms that may later spread to whoever contacts with the surface.

Research suggests that current protocols are not meeting the recommended microorganism load which may be contributing to the current incidence of surface acquired infections [3]. In reasons of these considerations, it appears that the introduction of self-disinfecting surfaces is very desirable. In this respect, different approaches for surface functionalization or modification to reduce contamination have been recently investigated, including approaches based on the development of surfaces with anti-adhesive properties, the incorporation of antimicrobial substances or the modification with biological active metals [4].

In the current paper, we follow indeed the latest strategy, since it appears particularly interesting because the obtained material has an appropriate balance between the antimicrobial and mechanical properties for a number of engineering applications.

For many industrial produced materials, one of the key problems is indeed to achieve optimal surface properties of components.

For this purpose, surface engineering techniques are frequently used which, in addition to minimizing the costs for the use of high-performance materials, allow to obtain the combined properties of the surface part and those of the substrate [5].

One of the most used techniques for optimizing materials in engineering applications (i.e., biomedical devices, pharmaceutical and food packaging machines) is the anodic oxidation, an electrochemical conversion process feasible on aluminum alloys aimed at producing a hard and adherent oxide layer [6, 7].

In this respect, aluminum and its alloys possess a variety of attractive properties (low density, high specific strength, high electric and thermal conductivity, low costs in relation to other light metals, high workability, no health hazard, recyclability) that rend very attractive for industrial application [8].

Aluminum alloys are increasingly used in many industrial fields as substitute for other materials. Specifically, aluminum can be treated by anodic oxidation in order to obtain a remarkable increase in surface hardness, abrasion, and corrosion resistance.

The resulting anodic oxide layers possess indeed some crucial advantages, including the strong connection to the substrate material forming a new material with unique properties: protection against corrosion and wear, insulation, thermal resistance, biocompatibility and, last but not least, antimicrobial properties [9, 10].

In order to explore and expand the potential of aluminum alloys (i.e., especially towards the development of self-disinfecting materials), the present paper describes the manufacture, the tribological, anticorrosive, and antimicrobial performances of a new material obtained by an innovative anodic oxidation, defined as golden hard anodizing G.H.A.®, shortly defined throughout the paper as “GHA.”

The anodic oxide surface thanks to the nanoporous structure can in fact be used as reservoir of substances (i.e., neutral or ionic) with lubricating and antimicrobial properties. Specifically, the GHA treatment involves filling the pores, created during the electrochemical anodization process, by silver ions (Ag^+^) [6]. The presence of Ag^+^ ions in the outer anodic oxide layer of GHA is particularly important since it increases the resistance to corrosion and abrasion and confers to GHA antimicrobial properties [11]. This property allows GHA to be proposed for a number of applications including industrial machines, kitchen utensils, heat exchangers, electro-medical, air and liquid filters, elements and finishes for hygiene (summarized in Table 1).

**Table 1:** Properties and applications of GHA as emergent materials

Properties	Applications
Low coefficient of friction, self-lubrication and resistance at consumption	Components of industrial machines
Corrosion resistance	Automotive components
High thermal conductivity and high thermodynamic efficiency	Components of office machines
High antistatic capacity	Kitchen items and appliances
Ability to absorb heat and return it as infrared waves	Components for housing and related accessories
High antibacterial capacity and anti-mold	Components for electronics and semiconductor
	Thermal radiators, heat exchangers, solar panels
	Clothing, electric blankets, carpets
	Filters for air conditioners

The antimicrobial surface of GHA is particularly relevant nowadays since the high rate of infectious disease outbreaks and illnesses stemming from SARS-CoV-2 virus have and will always be of great concern.

## Experimental

### Materials

The anodic aluminum oxide (AAO) and the gold hard anodized (GHA) treatment were applied on the following, commercially available, samples. EN-AW4006 (Al-Si) aluminum alloy plates, in form of disk (75 mm in diameter, 4 mm thickness) were used for the wear tests; EN-AW6082 (Al-Mg-Si) aluminum alloy in form of squared plates (50 mm × 50 mm, 10 mm thickness) were used for the evaluation of antimicrobial effect; rectangular plates (20 mm × 48 mm, 5 mm thickness) were used for the corrosion tests.

### Preparation of anodic oxide and gold hard anodized materials

AAO and GHA materials were prepared as follows: AAO was obtained by the type III anodization procedure, using sulfuric acid as the electrolyte [12]. GHA was obtained in accordance with EP 1980651A2 [13]. Notably, for AAO, the temperature of the electrolyte bath was maintained between -10 and 10°C and the current density set between 2.0 and 5.0 A/dm^2^, whereas, in the case of GHA, the temperature of the electrolyte bath was kept between -10 and 25 °C and the current density set from 1 to 10 A/dm^2^. The electrolyte bath contained a 20% (v/v) concentrated H_2_SO_4_ in water.

### GHA characterization

#### Roughness determination

The surface properties of GHA were analyzed by a roughness determination, following the ISO 4287 standard [14]. The GHA disk samples (75 mm in diameter, 4 mm thickness) were tested by a non-contact 3D optical profilometer, using a green light and 20× objective CCI-Lite Talysurf (Taylor Hobson, Leicester, UK). In each disk, 4 independent rectangular areas (20.04 mm × 0.83 mm) where identified for the profilometric analysis, each of them represented by a rectangle, measuring 20.04 × 0.83 mm. The analysis was performed by the software MountainsMap 7 (Digital Surf, Besancon, France) using the following operators: original reference image, spatial filter, fill in non-measured point filter, form removal filter, 3-D surface reconstruction of the analyzed area and extracted profile. Before the analysis, each sample has been cleaned with ethyl alcohol to remove any traces of surface dirt. The centre of each disk has been identified and marked so that it can be positioned under the profilometer light in the same point before and after the wear tests. For each disk, starting from the center, 4 roughness measurements were carried out. The surface topography was quantified by obtaining different geometric descriptors. Specifically, the roughness was expressed by the following parameters: Rz, Ra, Rq, Rsk, and Rku, where Rz is the maximum height of the profile, defined on the sampling length; Ra is the arithmetic mean deviation of the assessed profile, defined on the sampling length; Rq is the root mean square deviation of the assessed profile, it corresponds to the standard deviation of the height distribution, defined on the sampling length; Rsk is the skewness of the assessed profile, it represents the asymmetry of the height distribution, defined on the sampling length. The Rsk parameter is particularly important as it gives information on the morphology of the surface texture. Positive values correspond to high peaks spread on a regular surface (distribution skewed towards bottom) while negative values are found on surfaces with pores and scratches. Rku is the kurtosis of the assessed profile, representing the sharpness of the height distribution, defined on the sampling length; specifically, a surface with perfectly random height distribution has Rku equal to 3. For surfaces with few high peaks and low valleys, Rku is less than 3, while for surfaces with many high peaks and low valleys, Rku is greater than 3.

#### Hardness tests

The tests were conducted on the specimens employed for the pin-on-disk test, namely 100Cr6 steel balls (10 mm in diameter) and GHA disks (75 mm in diameter). The tests were performed with a Vickers FM micro durometer (Future-Tech Corp., Kawasaki, Japan). During microhardness testing, the Vickers square-based diamond pyramid indenter was pressed onto the material’s surface with a penetrator and a load of 1000 g load for steel balls and 50 g load for the GHA disks. The tests were performed by applying the load for 15 s. The diagonals of the resulting indention were measured under a DMI8A Leica optical microscope (Leica, Wetzlar, Germany), then the measurements and the test load were used in a specific formula to calculate the Vickers hardness value. Different number of tests was conducted at room temperature: in the case of balls (*n*=5) and in the case of disk (*n*=20).

#### Pin-on-disk tests

Wear tests were carried out, without lubrication, using the TR-20LE tribometer (Ducom Instrument Europe, Groningen, The Netherland) in a pin-on-disk configuration in accordance with the ASTM G99 standard [15]. In this configuration, the tested materials were analyzed under nominally non-abrasive conditions. The 100Cr6 steel balls (10 mm in diameter) were used as a counter body material and positioned perpendicularly to the GHA disks (the analyzed samples) lapped and ground. During the wear test, the disk rotated while the ball was pressed against the disk by a system of weights and levers. The ball was held fixed at a known position on the disk.

The tribometer was equipped with three sensors: a load cell for measuring the frictional force, a linear variable differential transformer (LVDT) sensor for measuring wear and an encoder for measuring the rotational speed of the disk. In particular, the LVDT sensor measures wear as positive displacement, if there is disk wear, or negative if there is the formation of a third body between the ball and the disk. In this way, it is possible to have a continuous measurement of the coefficient of friction (COF) during the test. The calculation of the wear rate of both the disks and balls was determined by weight-loss method; therefore, the disks and the balls, after ultrasonic cleaning for 5 min in acetone, were weighed with a Mettler Toledo AE 200 balance (sensitivity ± 0.00001 g) (Columbus, Ohio, USA), before and after the wear test. To determine the macroscopic characteristics of the disk wear tracks, stereo-microscope images were taken.

The tests were performed on GHA samples with an anodized layer thickness of 50 μm. The following testing parameters were used: sliding speed = 0.1 m/s, load = 50 N and sliding distances = 20 m and 250 m. Temperature and relative humidity were kept constant at 20.6 ± 0.1°C and 22.9 ± 0.1 UR% respectively and were measured with a thermo-hygrometric control unit (Delta Ohm HD2101). Each test was conducted in triplicate.

#### 3-D optical profilometry, scanning electron microscopy, and XRD analysis

The disk area containing the wear tracks was imaged by the non-contact 3D optical profilometer. For each wear track, 3 measurements were carried out at different points of the extracted profile. Known the average value of the track area, it was possible to calculate the disk volume loss and the wear rate of the disk using the following formulas.

1 $$ \mathrm{disk}\ \mathrm{volume}\ \mathrm{loss}=2\pi \mathrm{RA} $$

2 $$ \mathrm{wear}\ \mathrm{rate}=\frac{\mathrm{disk}\ \mathrm{volume}\ \mathrm{loss}}{\mathrm{load}\cdotp \mathrm{sliding}\ \mathrm{distance}} $$

where *R* is the radius of the circular wear track, A is the area of the wear track [16].

To determine the wear mechanisms that occurred on the disk, a scanning electron microscopy (SEM) microphotographs were used. The microscopic analysis was performed using a EVO40 SEM (Carl Zeiss, Oberkochen, Germania) equipped with a LaB6 thermionic source with an electron acceleration voltage (EHT) equal to 20,000 kV. With respect to the SEM, sample preparation and analysis were performed by the following procedure. The micrographs were recorded in secondary electron imaging (SEI-SEM) and back-scattered electron (BSE-SEM) mode, with an electron acceleration voltage (EHT) equal to 20 kV. Secondary electrons provided the topographic contrast, while backscattered electrons provided the compositional contrast. After the wear tests, each disk was carefully cleaned with acetone to remove wear debris and dust from the wear track. The cleaned disk was transferred onto a stub, secured with a carbon adhesive tape. Since the anodic oxide is a nonconducting treatment, the examination of the disks was undertaken in low vacuum SEM mode to reduce the electric charge due to impingement of the electron beam at their surfaces. Given that the nonconducting treatment is unable to discharge the negative charge, the air is injected into the specimen chamber around the specimen surface area. This allows for charge-free imaging of nonconductive specimens without having to coat them with a conductive material. Through the microanalytical technique of energy dispersive X-ray spectrometry (EDS), it was possible to make maps of the elements present on the worn surface. X-ray mapping shows the elemental distribution in a two-dimensional plot: maps for all detected elements are displayed through multiple windows, which show the scanned area. The regions that appear bright are the regions where the considered element is concentrated. As a result, elements present in a sample are coloured maps of estimates of elements.

Phase compositions of the wear debris were determined using a D8 ADVANCE (Bruker, Billerica, Massachusetts, USA) X-ray diffractometer with Cu-Kα radiation (λ = 1.5406 Å) and 2θ range from 6° to 120° with a step length of 0.02°. For structure solutions, the measurement time per step was 2 s. The X-ray diffraction (XRD) is based on X-ray interaction with the powder sample, the crystal structure of the powder was determined by Bragg–Brentano (θ-2θ) XRD measurements.

### Corrosion tests

Sealed GHA samples were subjected to accelerated corrosion tests in neutral salt spray (NSS) according to UNI EN 9227:2017 (standard which offers indications on the machinery for carrying out the test, the characteristics of the chemical solution and its use and three main types of tests) [17]. The corrosion resistance was performed using the Ascott model CC450 IP salt spray machine (Tamworth, UK). Specimens were continuously exposed to the artificial atmosphere constituted by spraying a 5% sodium chloride (w/v) solution at neutral pH (6.5-7.2), at a ratio of 1.5 ml/h. The tests were conducted at 35°C, for a total time of 1000 h, with intermediate observations performed at 360 and 500 h. Immersion specimens were placed in the same salt spray chamber, and nebulization was determined by two rain gauges with a diameter of 100 mm, corresponding to a collection surface of 80 cm^2^, positioned inside the test chamber. The temperature inside the chamber is controlled by using the Testo model 925 thermometer. Final observations were conducted after rinsing and drying the samples, by photographic imaging, without any manipulation of the GHA surface.

### Antimicrobial effect

The evaluation of the antimicrobial properties of GHA was performed following the ISO 22196:2011 standard [18]. As the procedure requires, two bacterial species were considered: a Gram-positive (*Staphylococcus aureus*) and Gram-negative (*Escherichia coli*). In addition, we have performed the test on other bacteria including *Legionella pneumophila*, *Pseudomonas auruginosa* and the fungi *Candida albicans*. The different microorganisms employed were used at a concentration of the starting inoculum ranging from 2.5 to 10 × 10^5^ CFU/l. The control (the non-anodized EN-AW6082 aluminum alloy) and the GHA samples had the common size standards in form of squared plates with the following dimensions: 50 mm ± 2 mm per side and < 10 mm of thickness. The control was tested in triplicate at time = 0 and time = 24 h. The GHA samples were tested in triplicate at time = 24 h. Each sample was placed in a sterile Petri dish, inoculated and then covered with a sterile polypropylene plastic film in order to spread the inoculum evenly over the sample surface and hold it in place. The samples were maintained at 35°C and a relative humidity of at least 90%. At the appropriate time, the neutralizing broth was added to each sample, placed onto a shaker and mixed thoroughly to facilitate the release of the inoculum from the sample surface. Serial dilutions of the obtained broth were plated. All plates were maintained at 35°C for 24-48 h. After incubation, the number of bacterial colonies were counted and recorded.

The results of the antibacterial effects are expressed in terms of antibacterial activity (*R*) [18]. The value of the antimicrobial activity was calculated according to the below reported formula and recorded as log reduction.

3 $$ R=\left({U}_t-{U}_0\right)-\left({A}_t-{U}_0\right)={U}_t-{A}_t $$

where *R* is the antimicrobial activity, *U*_*o*_ is the average of logarithm numbers of viable bacteria from the controls at time = 0 h, *U*_*t*_ is the average of logarithm numbers of viable bacteria from the controls at time = 24 h, *A*_*t*_ is the average of logarithm numbers of viable bacteria from GHA samples at time = 24 h. According to the standard, an antimicrobial effect is determined to have an effectiveness when the *R* is ≥ 2.0.

## Results and discussion

### Preparation of anodic oxide and gold hard anodized materials

Aluminum alloys are increasingly used as substitute for other materials (i.e., iron, steel); in fact, aluminum is lighter and has a better corrosion resistance than iron and steel. Moreover, anodic oxidation on aluminum alloys increases the surface hardness, abrasion, and corrosion resistance, and improves the application of aluminum (e.g., the automotive, biomedical, electrical engineering, household articles fields and many others).

Hard anodizing process is characterized by a careful control of the process parameters and allows obtaining protective coatings. These layers are characterized by a quite high surface roughness (a typical value is Ra = 2.5 μm) that it is possible to reduce by grinding. Hard anodic layers are mainly used for applications that need low-stress abrasion and corrosion resistance.

For the preparation of the anodic oxide and GHA, the following aluminum alloys were selected: EN-AW4006 (Al-Si) and EN-AW6082 (Al-Mg-Si) in form of disks, plates or rods.

Such alloys were chosen in reason of their good to optimal properties in terms of anodization ability and surface texture (i.e. smoothness).

**Fig. 1:**
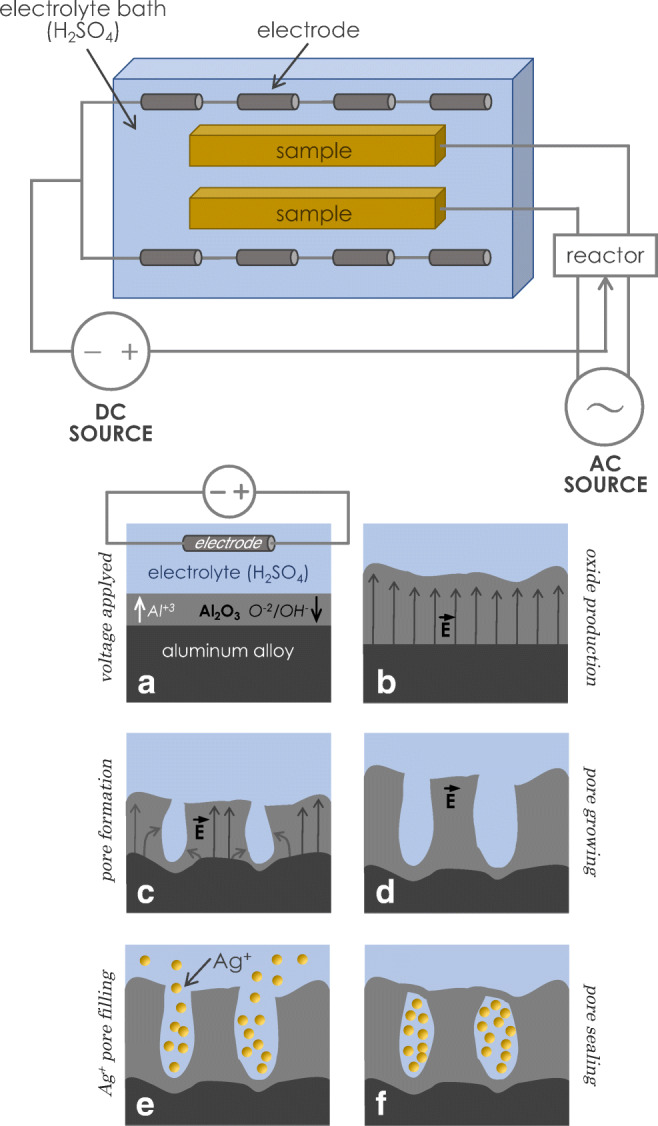
General scheme of the GHA oxidation process (upper panel) and pictographic description of the various phases of GHA production processes (lower panel). Formation of barrier oxide on the entire area (**a**); local field distributions caused by surface fluctuations (**b**); formation of pores by electrolytic dissolution (**c**); pore growth (**d**); pore filling by Ag+ ions (**e**) and finally sealing process by hot water treatment (**f**)

The procedure followed for the preparation of the GHA is summarized in Fig. 1 and in Table 2, where are summarized the main factors and the relative set values; briefly, for GHA, the apparatus for the electrochemical treatment includes a rectangular electrolytic tank, in which the electrodes are placed on both sides and connected in parallel. Each of the electrodes is composed, in turn, of four smaller units (constituted of carbon steel) and connected in series at regular intervals along the longitudinal direction. The samples to be treated (e.g., the aluminum alloy) are arranged between electrodes and immersed in the electrolyte (based on sulfuric acid) to which a silver salt is later added.

**Table 2:** Procedural steps for the preparation of GHA

Factor	Value (unit)
Electrolyte solution	Sulfuric acid (H_2_SO_4_, 20%, w/v)
Continuous current density	1–10 (A/dm^2^)
Voltage	20–100 (V)
Temperature	-10–+25 (°C)
Treatment time	2–4 (h)
Color	Gold brown to dark gray
Film thickness	5–100 (μm)

The electrochemical treatment is based on the application of a combination of an alternating current and a positive direct current. As shown in the scheme (upper part of Fig. 1), the positive pole of the DC source is connected to a reactor while the negative pole is connected to the electrodes. The reactor is also connected to an AC source, in this way there is a combination of currents which are supplied to the samples. As schematized in the lower part of Fig. 1, the initial part of the process is characterized by the formation of a thin oxide compact layer of Al_2_O_3_ (A), later the process involves the increase of the oxide thickness (B) and the initial pore formation (C); in a further phase the dimension of the pore (diameter and depth) continue to grow (D). The pores, formed in the oxide layer, start to be filled with Ag^+^ ions (present in the electrolytic bath) (E), finally the silver containing pores are sealed by treatment with hot water (F). N0tably, the layers formed by the electrochemical process are made up of a mixture of alumina and hydrated alumina, and are, as stated, highly porous. Pores are typically 200–350 nm in size and are oriented perpendicularly to the metal surface.

### GHA characterizations

The material surfaces are generally characterized by a number of different layers, namely, (1) a deformed layer, (2) a reacted layer, and (3) a contaminated layer. The characteristics of the plastically deformed layer depend on the material and on the manufacturing processes. Typically, in metals, a work-hardened layer is formed with possible local microstructural modifications. The reacted layer is formed spontaneously due to exposure to the surrounding environment. In metals, an oxide layer is formed after air exposure. In particular, by electrochemical process, on the surface of aluminum alloys grow a layer of compact amorphous Al_2_O_3_ oxide, covered by a thicker and porous layer of hydrate oxide. In order to characterize the produced GHA, specifically the anodized superficial layer, different procedures were undertaken, as described in the following sections.

#### Roughness determination

**Fig. 2:**
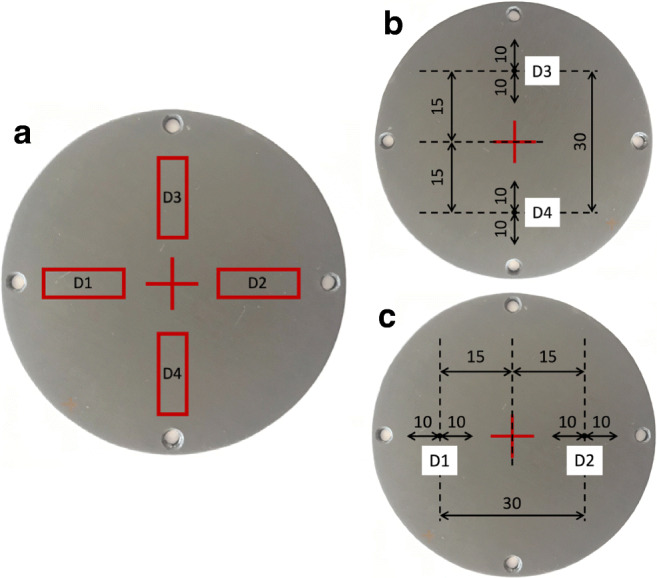
Geometrical constructs for the identification of the areas considered for the determination of the roughness of GHA specimens. Each area has a dimension of 20.04 × 0.83 mm

The areas investigated for the determination of the roughness are indicated in Fig. 2; notably, the same regions were later considered for the determination of the wear scar area (see 3.2.2 pin-on-disk tests section) [19].

**Fig. 3:**
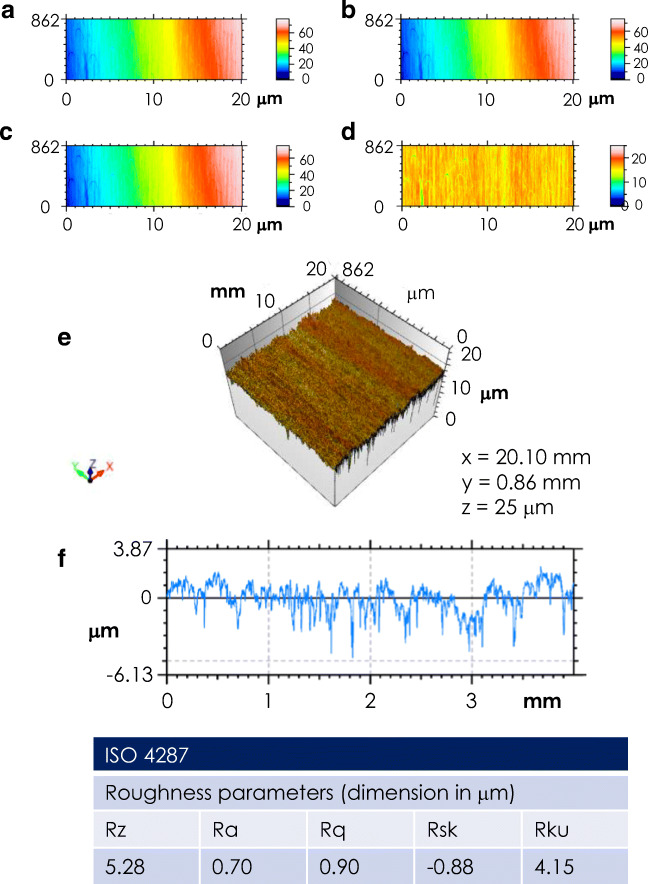
Representative profilometric analysis of the GHA surface topography for sample GHA50/20-1. **a**–**d** images obtained by application of different filters, namely, original reference image (**a**), spatial filter (**b**), fill in non-measured point filter (**c**), form removal filter (**d**). **e** 3-D surface reconstruction of the analyzed area. **f** Extracted profile. In the inset table are reported the data relative to the analyzed roughness and surface texture parameters obtained according to the ISO 4287 standard (the Gauss filter was set at 0.80 mm)

As exemplified in Fig. 3, the sample surface was imaged applying different filters. specifically, as reported in panel B–D (whereas panel A reports the row image without any filter applied). The image in panel B reports the matrix filtering of the surface, allowing to improve the images and search for details. Spatial filtering does not use the Fourier transform, but is made by moving a small filtering matrix (called a kernel matrix) over the surface. Specifically, the applied operator consisted in averaging each point with its 5×5 neighbourhood. Panel C reports the image obtained with the fill in non-measured points filter; it is used to rebuild non-measured points in the case when they are numerous and scattered. Panel D reports the image obtained by a form removal filter; such filter consists in approximating the general form of a surface using a mathematical function varying slowly, using a 4th grade polynomial. As further investigation of the surface characteristics, a 3D view was produced (Fig. 3, panel e); this view allows to study a 3D-representation and to extract the surface profile (see panel F). Finally, from the surface profile, the roughness parameters were derived (see inset table of Fig. 3).

**Fig. 4:**
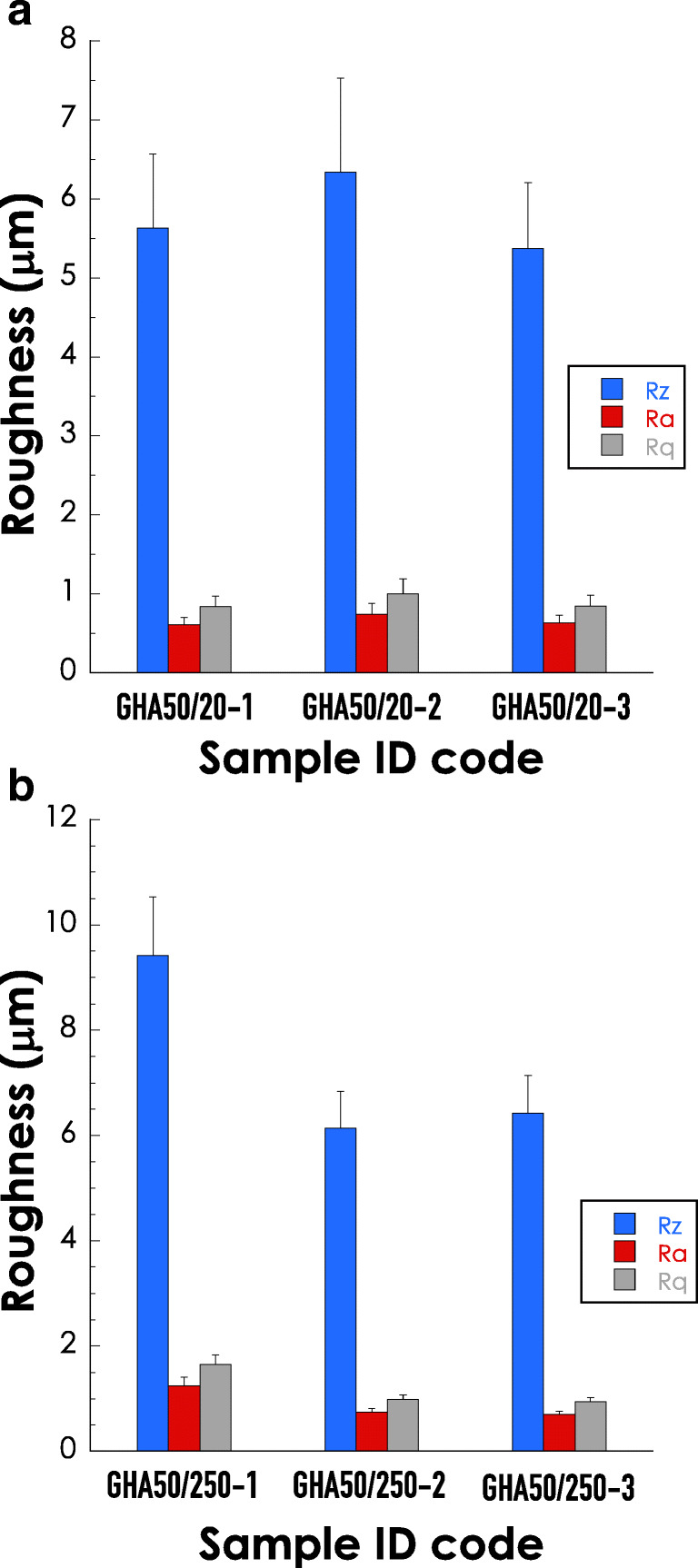
Summary of the profilometric parameters relative to the roughness of the indicated GHA samples. The determinations were performed before the wear tests. Blue bars (Rz), red bars (Ra), and grey bars (Rq). The data represent the average of 3 independent measurements ± SD

With respect to the surface parameters, as indicated in Fig. 4, all samples were characterized by a rather smooth surface comparable to a level of finishing, between grinding (Ra ≈ 0.8 μm) and lapping (Ra ≈ 0.2 μm). Regarding Rq, this parameter provides similar information with respect to Ra, but it is more sensitive to large deviation from the main line. Finally, data for Rz are particularly interesting since the low levels observed indicate that the samples do not present important protruding peaks that might affect sliding contact functions. In addition, it is important to underline that all samples were rather homogeneous in term of surface topography; no major differences were indeed observed for all the considered roughness parameters (see Fig. 4). Altogether, the surface features were therefore comparable in term of sample surface properties, allowing a correct statistical analysis of the further pin-on-disk tests.

#### Hardness tests

In order to gain information regarding the material properties of the base aluminum alloys and GHA Vickers hardness test was performed. In addition, the test was also conducted on the 100Cr6 steel balls later employed in the pin on disk tests. The results of the test are included in Table 3. As expected, the hardiness of the GHA is far higher than that of the starting alloy base (i.e., an increase of more than 10 times).

**Table 3:** Hardness value for aluminum alloy, GHA, and 100Cr6 steel ball

Specimen	Vickers hardness HV0.05	Vickers hardness HV1
Aluminum alloy (EN AW6060)	30	n.d.
GHA	385	n.d.
Steel ball	n.d.	781

Hardiness tests were performed following the indicated procedure as described in the experimental section. Values represent the average of 3 independent determinations

#### Pin-on-disk tests

As a prelaminar note, it is important to remember that wear tests on materials with an anodic oxide layer are usually carried on unsealed surface (i.e., with open pores); in this condition, the anodic layer presents a remarkably high hardness and great wear resistance, but on the contrary it has poor corrosion resistance (with respect to oxide layers with sealed pores).

Since for the final uses (e.g., food and pharmaceutical packaging, mechanical, naval, etc.), a high corrosion resistance is considered mandatory, and all the GHA specimens present a sealed pore surface.

Moreover, it is to be considered that corrosion and wear resistance are two faces of the same coins. In operation, an anodized component that corrodes will wear prematurely and, if it wears prematurely, it will tend to corrode more easily. Therefore, in the current paper, the tribological tests were performed in conditions of real use, therefore considering only sealed pore surface specimens.

**Fig. 5:**
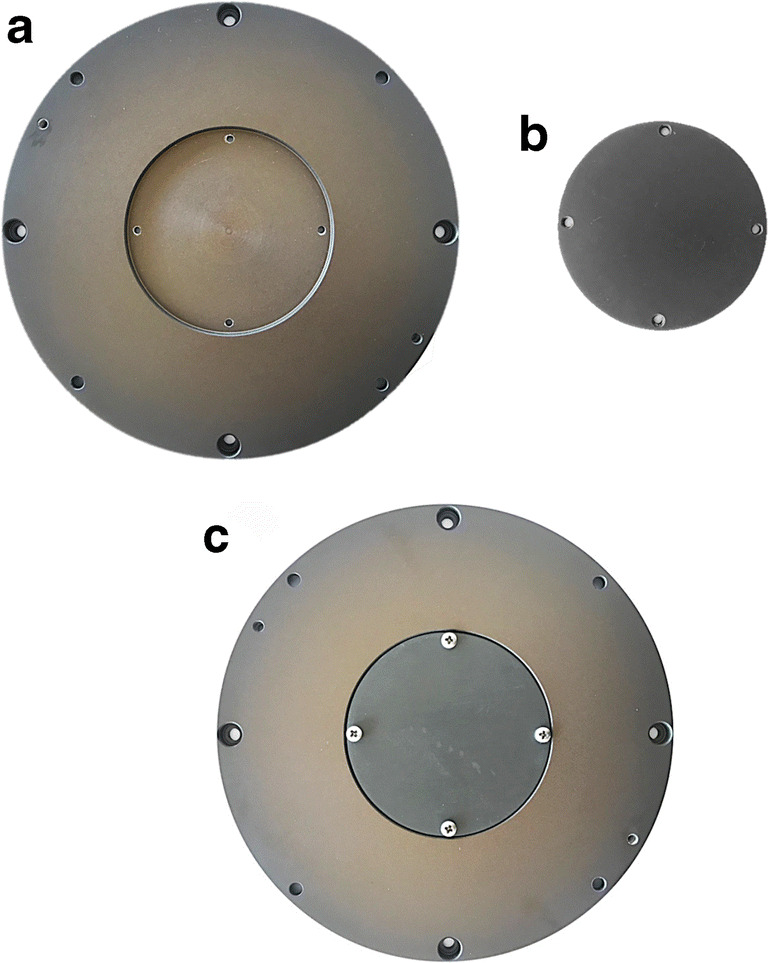
Wear test sample setup. **a** sample holder (diameter 165 mm), with a central cavity for the sample; **b** GHA sample in form of a disk (diameter 75 mm); **c** view of the sample holder with the inserted sample

As previously discussed, the roughness of the discs was measured before the wear tests. Pin on disk tests were conducted using a specific set up as depicted in Fig. 5 constituted of a sample holder (diameter 165 mm), with a central cavity for the GHA sample in form of a disk (diameter 75 mm) and a stationary counter body represented by the 100Cr6 steel ball (diameter 10 mm). Therefore, given the different curvature of the surface of the disc and the end of the pin, the two bodies have, ideally, only one point of contact. In particular, the contact area is characterized by a first elastic (Hertzian) and then plastic contact analysis.

Before the wear test, the contact between the two bodies is of a non-compliant type with parabolic distribution of the pressure within the contact area and the heat flow due to friction. However, it should be considered that the Hertzian stresses and pressures are valid only at the beginning of the test [20]; in fact, due to the effect of the external load, the two components begin to deform plastically and the contact between pin and disk is progressively enlarged, leading to an increase in the effective contact area and a decrease in the pressure contact. Therefore, it is appropriate to consider that in the contact area there is a uniform distribution of the pressure determined by the hardness of the less hard of the two contact materials [21].

**Fig. 6:**
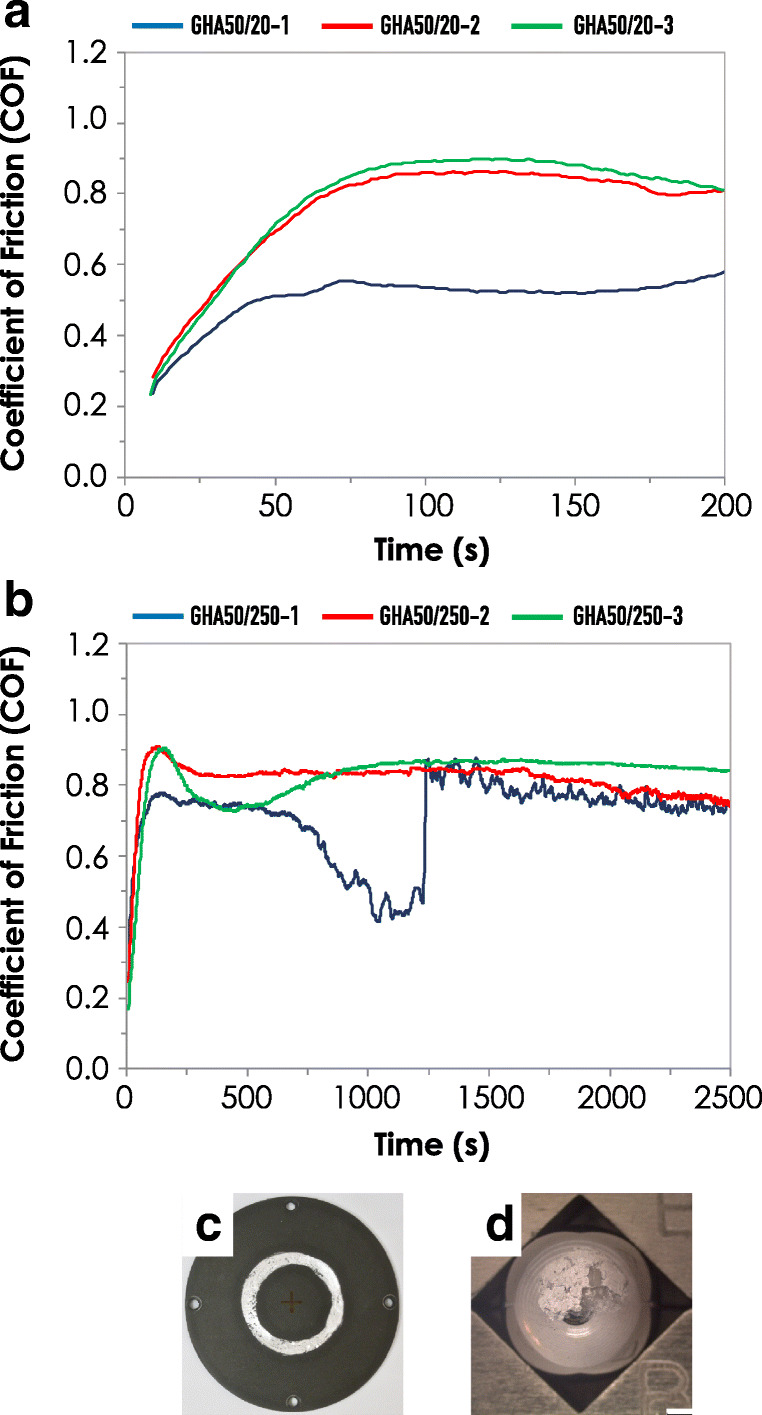
Analysis of the coefficient of friction (COF) vs time. Determinations were performed at a speed of 0.1 m/s and a load of 50 N. **a** COF determined with a sliding distance of 20 m on different GHA samples, blue line (GHA50/20-1), red line (GHA50/20-2), and green line (GHA50/20-3). **b** COF determined with a sliding distance of 250 m, blue line (GHA50/250-1), red line (GHA50/250-2), and green line (GHA50/250-3). Measurements were conducted in triplicate; each line represents the trend of an independent sample

The results of the pin on disk tests are reported in Fig. 6. Data are expressed as relationship between time to COF for GHA samples analyzed at 2 different sliding distance, namely 20 m and 250 m. For each sliding distance, 3 independent samples (i.e. disks) were considered.

From the analysis of the reported graphs, it is evident that the curve fits indicate that the samples were substantially uniform, even if with few exceptions, in term of wearing resistance. In Fig. 6a, the lower COF values found for the sample GHA50/20-1 were attributed to a possible variation in the formation of the tribolayer, together with a slight difference in the hardiness of the steel ball. In the case of Fig. 6b, the different behavior of sample GHA50/250-1 was, on the contrary, attributed to the higher initial roughness of the sample. Indeed, Rz, Ra and Rq are slightly larger (see Fig. 4) with respect the other 2 analyzed samples, resulting in a markedly different wear track (Fig. 6c), with a much larger track and exposing the base aluminum alloy and formation of third body on the surface of the steel ball (see Fig. 6d).

**Fig. 7:**
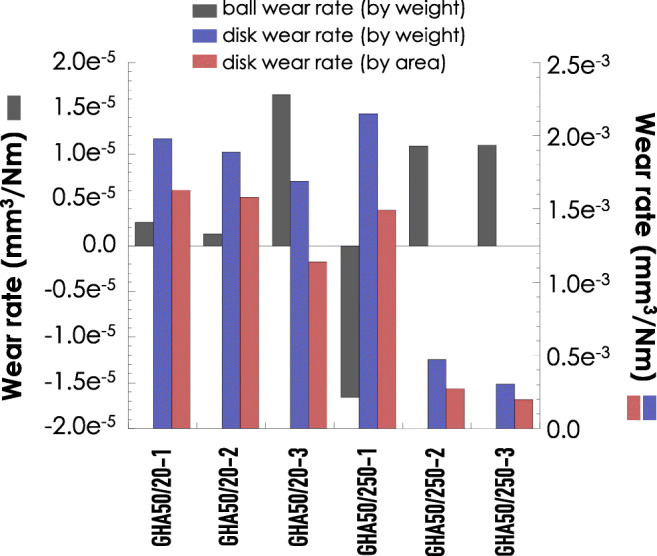
Wear rate of both the disks and balls as determined by weight-loss method. Red circles refer to the GHA insert weight loss, whereas the blue circles refer to the balls weight loss

After pin on disk test, the involved specimens were further characterized for tribological aspects. In detail, as reported in Fig. 7, both the disks and balls were analyzed for determining their wear rates. Notably, two alternative methods were used: the weight loss method and the method involving the area of the scars. From the reported data, the following conclusions can be withdrawn: (a) as expected by the hardness tests, the wear rate of balls is always very small when compared to that of disks (about 2 order of magnitude); (b) the data relative to the wear rate by weight are always larger than those obtained by the area method; (c) increasing the sliding distance (from 20 to 250 m) causes a relevant increment of the ball wear rate whilst for the disk a lower rate was observed, and this latest feature was attributed to the formation of a tribolayer on disk surface [22].

#### 3-D optical profilometry, scanning electron microscopy, and XRD analysis

**Fig. 8:**
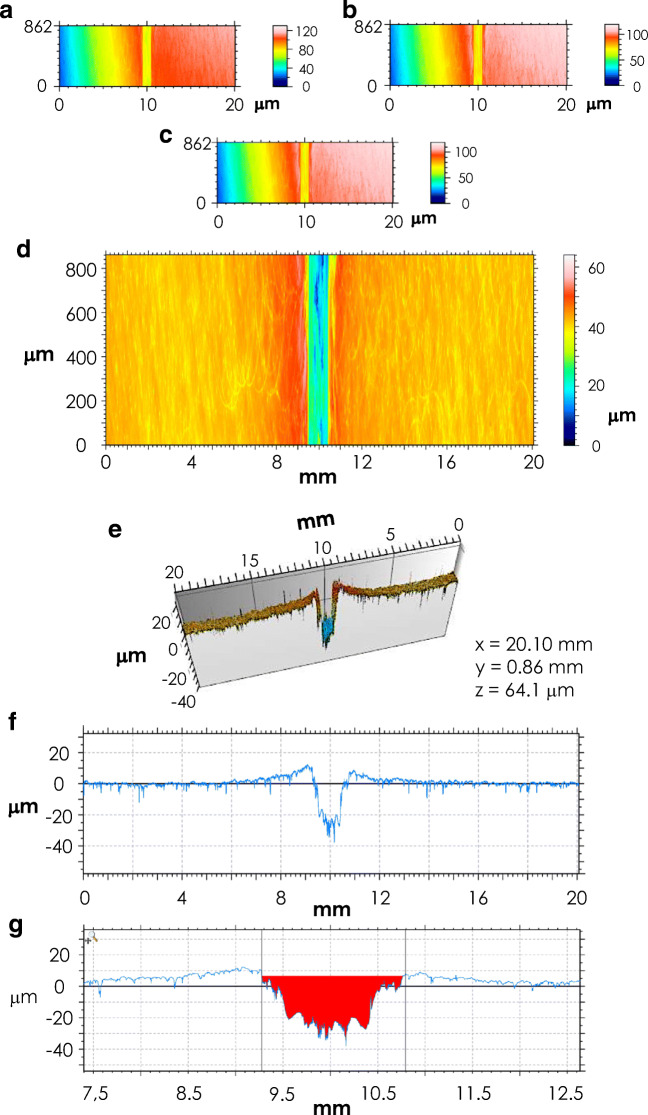
Representative profilometric analysis of the wear track. **a**–**d** Images obtained by application of different filters, namely: original reference image (**a**), spatial filter (**b**), fill in non-measured point filter (**c**), form removal filter (D). **e** 3-D surface reconstruction of the analyzed area. **f** Extracted profile. **g** Area measure of the wear track

In Fig. 8, a typical example of a wear scar analysis is reported. In detail, the scar area was imaged applying the filters used for the determination of the roughness (see paragraph 3.2.1 and Fig. 3). In panel E of Fig. 8, a 3-D surface reconstruction of the analyzed area (the wear scar) is reported, together with the extracted profile and the measure of the area of the wear track, highlighted in red (panels F and G respectively).

**Fig. 9:**
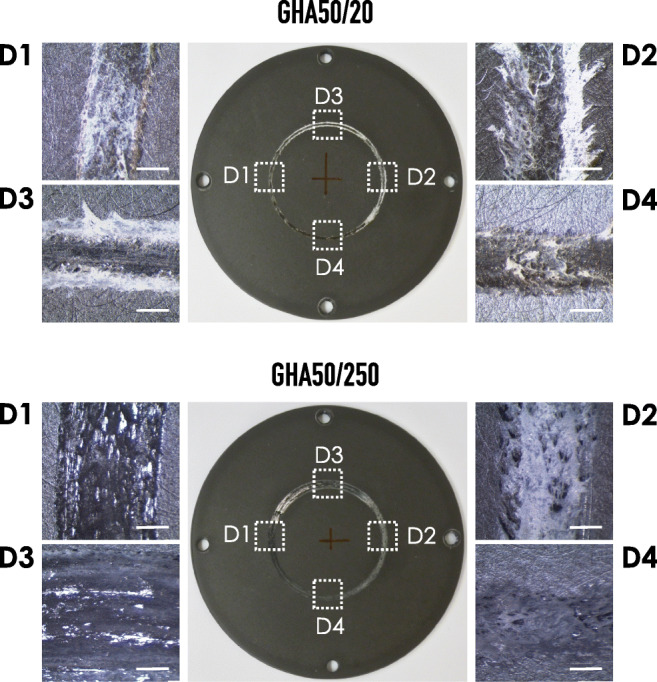
Representative optical stereomicrographs of the wear track after wear test. Images are relative to the sliding distance of 20 m (sample GHA50/20) and 250 m (sample GHA50/250). For each sample, 4 independent areas (indicated by the dotted line rectangles) were imaged. Bars correspond to 0.5 and 2.0 mm for sample GHA50/20 and GHA50/250, respectively

As further investigation of the wear surface, an optical stereomicroscopic analysis was performed. As reported in Fig. 9, four different areas were considered (D1, D2, D3, and D4), specifically identified by dotted squares. The reported images demonstrate, as expected, that the width of the wear track is related to the sliding distance and 250 m track are indeed much larger than those resulting from a run of 20 m. In addition, the images indicate that at 20 m (sample GHA50/20) the plastic deformation is predominant, especially in the outer region of the track. This behavior was attributed to the thin layer of oxide tribolayer that is thickened later, as evident from the images taken in the samples run with a 250 m sliding distance (sample GHA50/250).

**Fig. 10:**
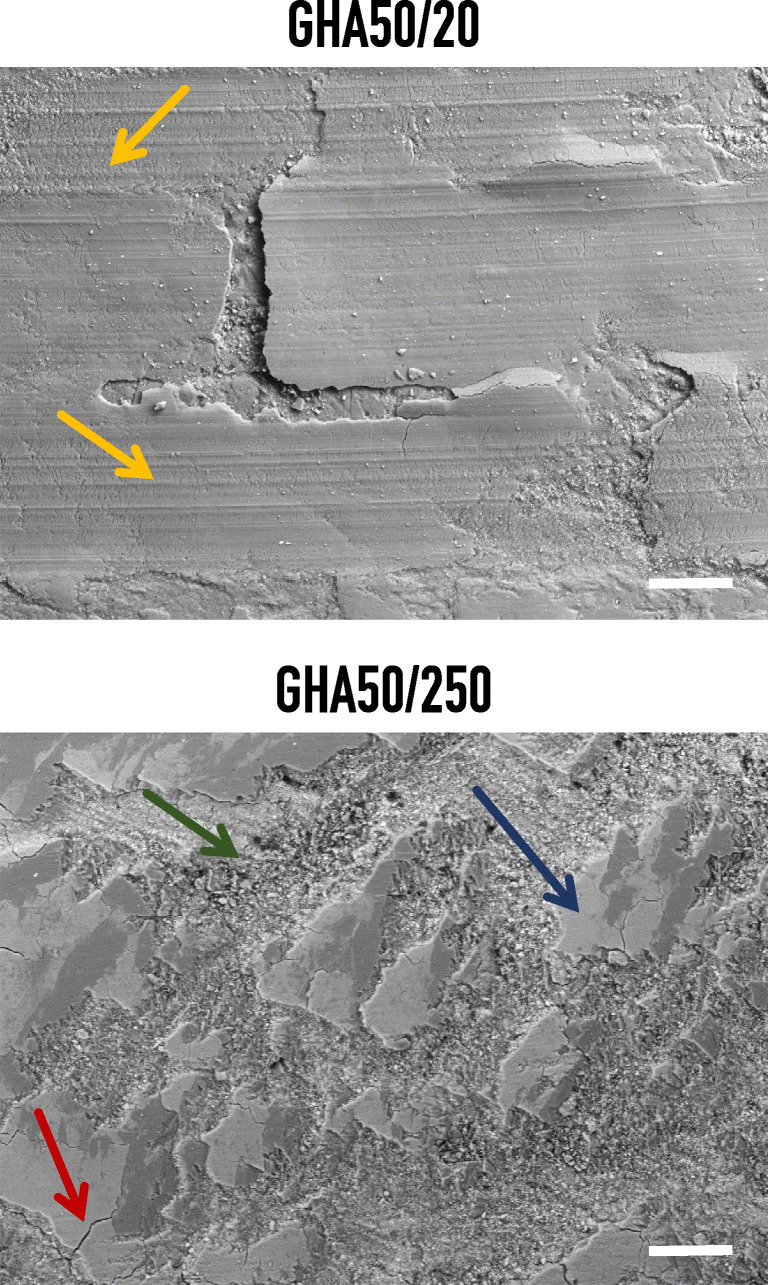
Representative SEM microphotograph of a GHA sample after wear test performed with a sliding distance of 250 m. The imaged area corresponds to the wear surface. GHA50/20 yellow arrows: abrasions. GHA50/250 blue arrow: tribological layer; red arrow: oxide fractures; green arrow: debris. Bar corresponds to 100 μm

**Fig. 11:**
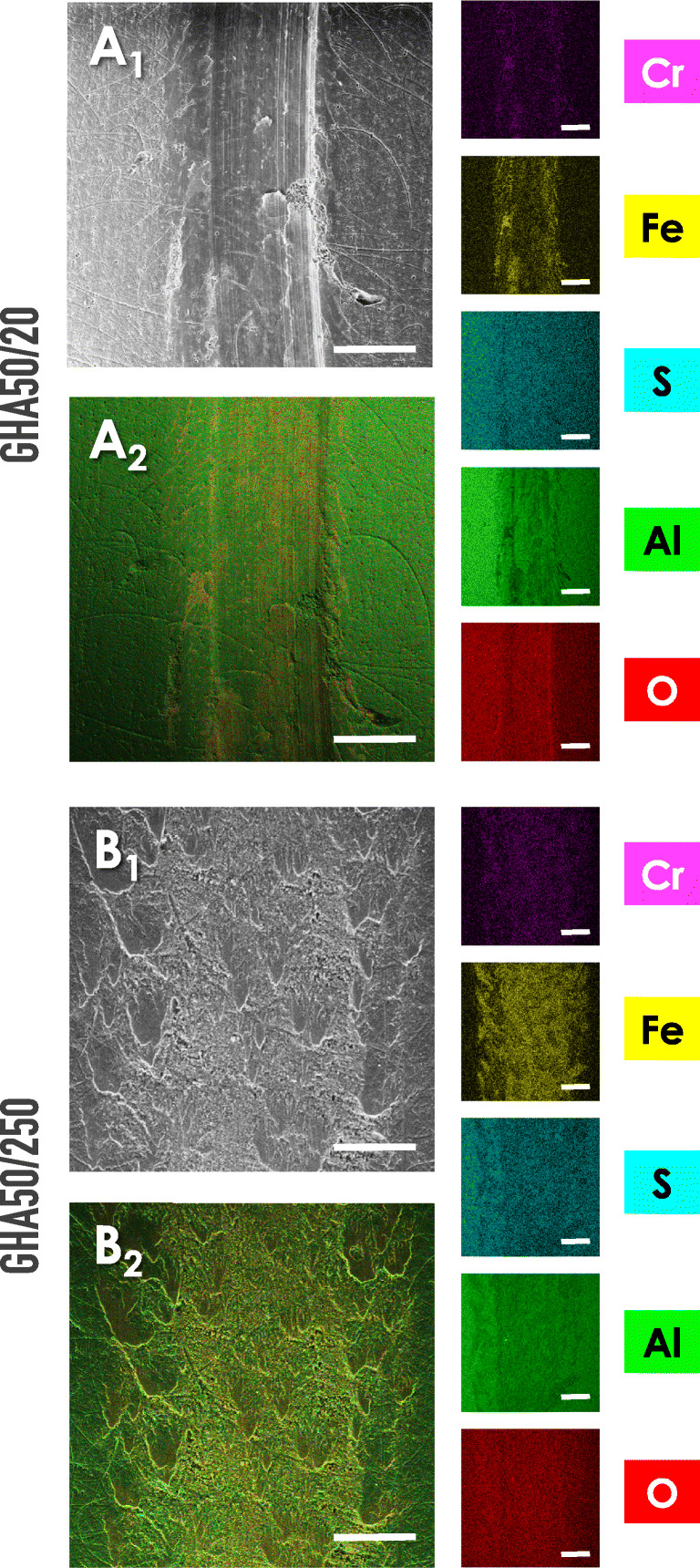
Representative images relative to the SEM/EDS map analysis performed on the indicated GHA samples, at the sliding distance of 20 m (sample GHA50/20) and 250 m (sample GHA50/250). Determinations were performed after the wear tests. A_1_-B_1_: SEM microphotography captured with the back-scattered electron (BSE) modality; A_2_-B_2_: SEM/EDS map. Small panels report the image relative to the EDS analysis for the indicated elements. Bar corresponds to 500 μm

In order to have a detailed investigation on the wear surface, SEM and SEM/EDS analyses were undertaken, as reported in Figs. 10 and 11.

As evident from the image reported in Fig. 10, the main wear mechanisms, on a microscopic scale, were abrasion and tribo-oxidation. Tribo-oxidative wear was attributed to the interaction of surfaces with the atmosphere oxygen and is therefore given by a combination of oxidative and mechanical actions in the contact area between body and counterbody.

Particularly at 250 m (sample GHA50/250) the wear is characterized by the formation of surface oxide scales that reduce the contact between the two bodies and act as a sort of solid lubricant, reducing friction.

The mechanism of tribo-oxidation was attributed to the high contact temperature caused by loading and sliding conditions that are particularly intense; such conditions result therefore in the formation of a tribological layer (i.e., the surface oxide scales indicated by the blue arrow) that is formed due to the compression of the 2 bodies. When the critical thickness of the layer is reached, the oxide fractures orthogonally (fractures are indicated by the red arrow in Fig. 10) to the flow direction giving rise to debris (indicated by the green arrow in Fig. 10) that can remain in the contact region or leave the tribological system.

Differently, the samples exposed to short sliding distance (GHA50/20) present a different wear mechanism, predominated by an abrasive wear (indicated by the yellow arrow in Fig. 10). This phenomenon takes place by a three-body abrasion, causing a plastic deformation of the disk surface (the represents the softer material).

A confirmation of this tribological analysis is represented by the SEM/EDS maps reported in Fig. 11. From the analysis of the maps, the following indications can be withdrawn. (a) In the GHA50/20, the content of Cr and Fe is lower than that evidenced in the GHA50/250. (b) The wear track width is much larger in the GHA50/250 with respect to GHA50/20 (confirming the stereomicrographs reported in Fig. 9). (c) The oxygen is uniformly present on the entire surface of the samples; this indicates that the anodic oxide layer covers the surface without exposure of the underneath aluminum alloy substrate. (d) The uniform presence of sulphur is due to the use of H_2_SO_4_ in the electrolyte bath employed for the preparation of GHA. The non-presence of Ag as a chemical element on the EDS spectra has been attributed to the misidentification of the Al Kα coincidence as Ag Lα of the minor/trace content of Ag with respect to the bulk Al.

**Fig. 12:**
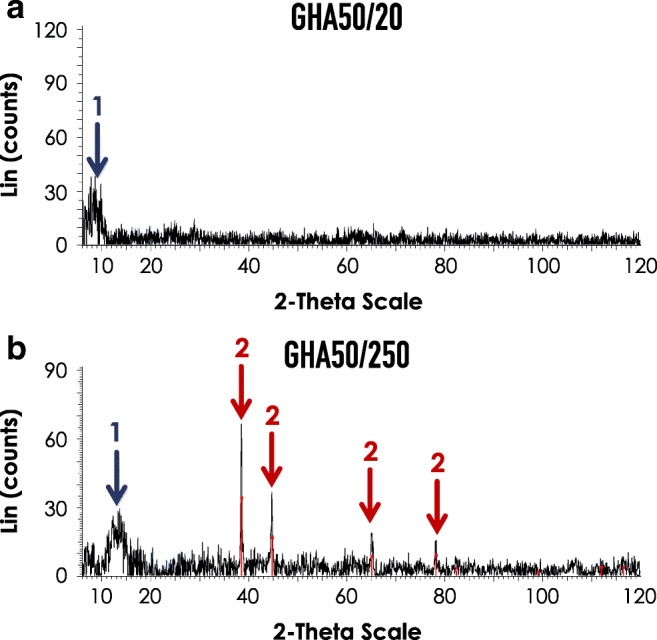
X-ray diffractometric spectra (XRD) of the powder collected after the wear tests. 2 independent samples were considered sample GHA50/20 (**a**) and sample GHA50/250 (**b**). Arrow 1 = broad peaks, attributed to the amorphous phase (aluminum in form of AlO^.^OH). Arrow 2 = sharp peaks, attributed to the crystalline phase

As further characterization, a XRD analysis on the wear debris was performed. The spectra reported in Fig. 12 indicate the presence of an amorphous phase (aluminum in form of AlO^.^OH) in all the samples (broad peak indicated by arrow 1), whereas crystalline phase peaks (evidenced by sharp peaks, arrow 2) are present only in the powder obtained from samples with a sliding distance 250 m. This behavior was attributed by the fact that at longer sliding distance, traces of debris derived from the aluminum alloy substrate are detectable.

### Corrosion tests

Salt spray tests were performed to detect discontinuities, pores, defects, and damage in the GHA samples and for quality control purposes. Specifically, neutral salt spray test (NSS) was carried on following the procedures that are commonly applied to metals, their alloys, and anodic oxide-treated alloys. The performed test allows to obtain accelerated results, since 1 h of exposure corresponds to about a week of exposure to an atmosphere free of particularly polluting agents.

**Fig. 13:**
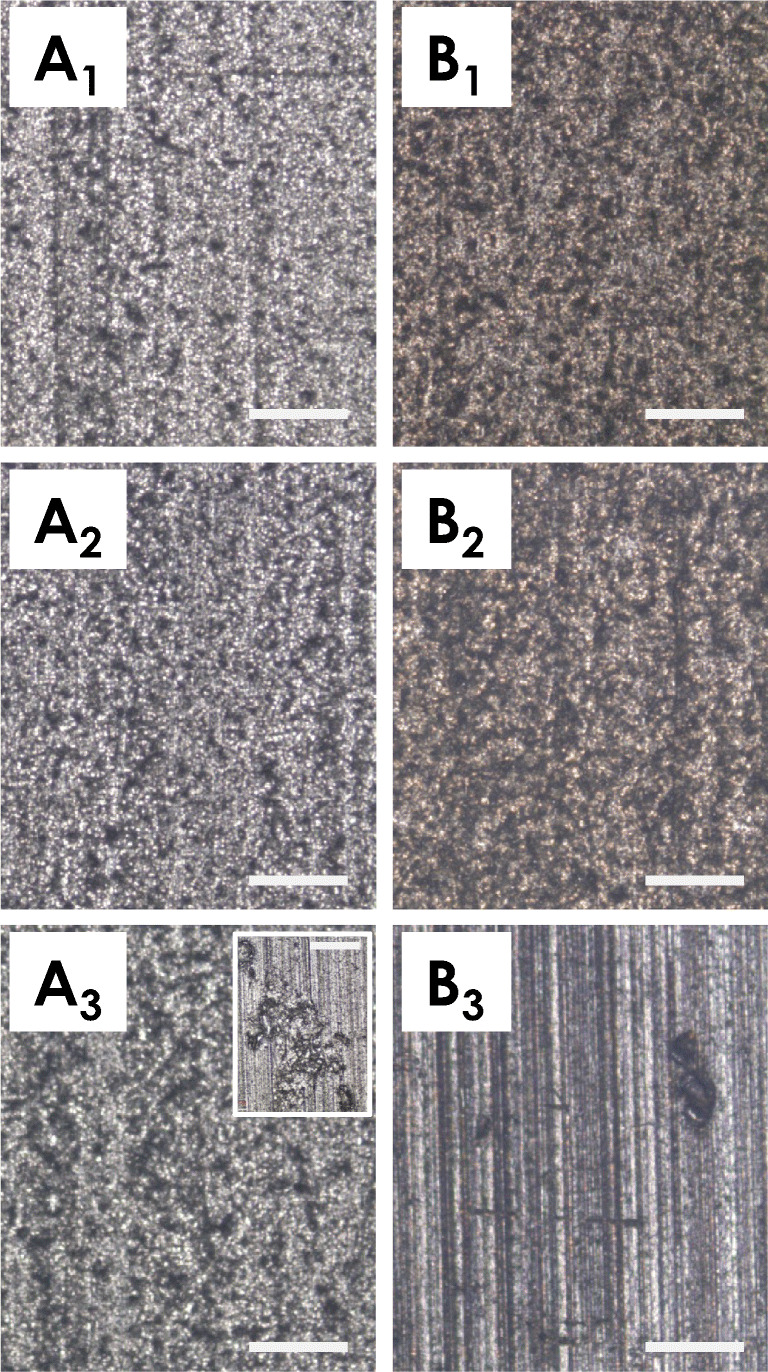
Representative optical microscope images relative to the corrosion tests in NSS. Anodic oxide (**a**) and GHA (**b**) samples were imaged at *t*=0 h (1), at *t* = 500 h (2), and at *t* = 1000 h (3). The inset in panel A_3_ reports a magnification of a region presenting a crater formed by corrosion pitting. Bars correspond to 250 μm

The NSS tests are summarized in Fig. 13; the surface morphology of GHA specimens (panels B) was compared to that of anodic oxidized Al alloy without silver ions, used as control counterpart (panels A).

The experiments were conducted for different length of time: *t*=0 h, *t* = 500 h, and *t* = 1000 h. The obtained results indicated that no significant differences were detectable after 500 h of treatment. Whereas, after 1000 h, the surface of the control material presents a general corrosion, with large corroded areas, in addition a number of pits are clearly visible (see inset of Fig 13, A_3_). The obtained results, therefore indicated that the presence of Ag^+^ ions have a beneficial effect on the corrosion resistance of the GHA.

### Antimicrobial effect

In order to obtain information on the possible antibacterial activity of GHA, specific tests were performed following the international standard procedure reported in the ISO 22196:2011 [18]. Notably, the antimicrobial effect of GHA was always compared to that of the same material without the presence of silver in the outer anodic oxide layer, used as control.

To define the antimicrobial activity, the value *R* was employed, where a value of *R* ≥ 2.0 indicates a significant bactericidal activity.

**Fig. 14:**
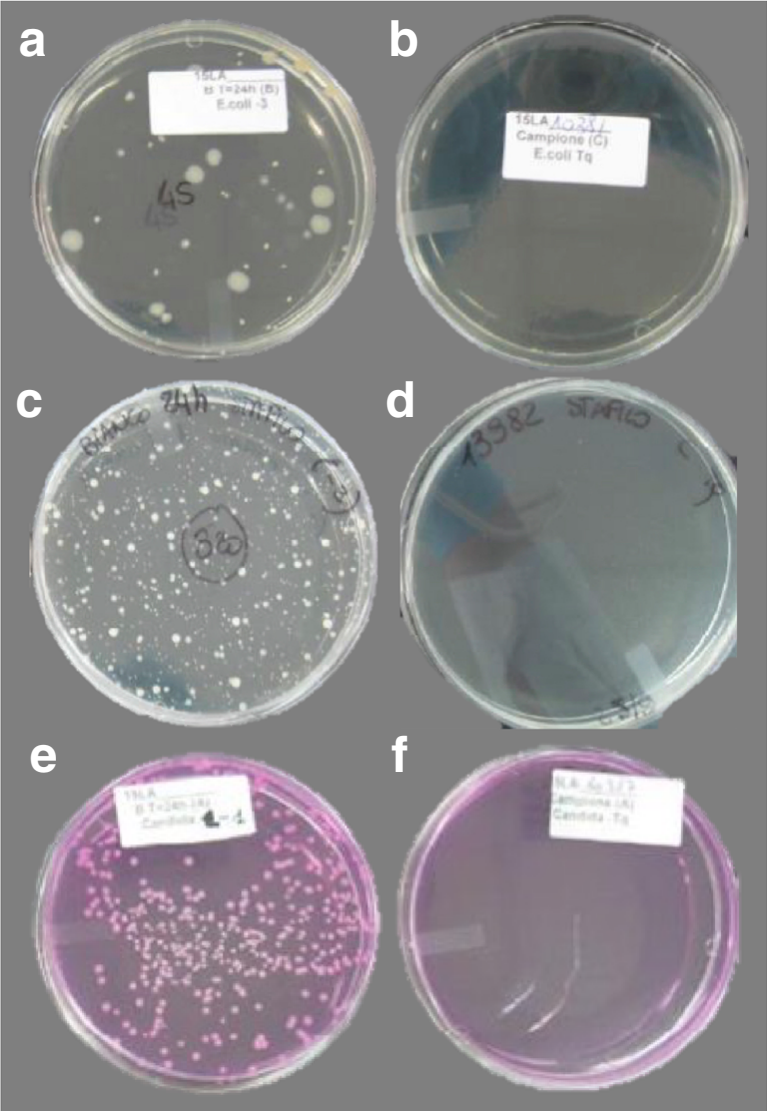
Antimicrobial activity of GHA as determined by ISO 22196:2011. The imaged petri dishes refer to the following samples after 24 h from the inoculation. **a** *Escherichia coli* (control, untreated), **b** *Escherichia coli* (in the presence of GHA), **c** *Staphylococcus aureus* (control, untreated), **d** *Staphylococcus aureus* (in the presence of GHA), **e** *Candida albicans* (control, untreated), **d** *Candida albicans* (in the presence of GHA)

Following the parameters of analysis and canons of determination of the used standards, the GHA samples showed an appreciable bactericidal efficacy, showing *R* values spanning from 3.6 (in the case of Escherichia coli) to 4.2 (in the case of Staphylococcus aureus); data are summarized in Table 4 and Fig. 14. The *R* values reported in Table 4 are well supported by the analysis of the petri dishes, that clearly show, that in the case of GHA samples, there are no visible colonies for all the testes microorganisms: *Escherichia coli* (Fig. 14b), *Staphylococcus aureus* (Fig. 14d), and *Candida albicans* (Fig. 14f).

**Table 4:** Determination of antibacterial activities on GHA samples

Bacteria tested	*R*	Antibacterial activities (% of abetment)
*Escherichia coli*	3.6	> 99.99
*Salmonella typhimurium*	3.3	> 99.99
*Staphylococcus aureus*	4.2	> 99.999
*Pseudomonas aeruginosa*	2.6	> 99.9
*Legionella pneumophila*	2.9	> 99.9
*Candida albicans*	3.1	> 99.99

Values represent the average of 3 independent determinations

The bactericidal activity considering different thicknesses of treatment (10 μm, 25 μm, and 40 μm) was also tested (see Table 5). The determination of antibacterial activity remained constant for each tested strain, indicating that the bactericidal activity of GHA is linked to the treatment itself (i.e.. the presence of Ag ions).

**Table 5:** Determination of antibacterial activities (*R*) on GHA samples with different thicknesses

Antibacterial activities (R)			
GHA samples	*Escherichia coli*	*Staphylococcus aureus*	*Candida albicans*
10 μm thickness	3.3	4.2	3.2
25 μm thickness	3.6	4.2	3.1
40 μm thickness	3.3	4.2	3.2

Notably, the antimicrobial activity of GHA was not tested limiting to the typical bacteria used in this kind of test, but spans on other important microorganisms such as bacteria responsible for diseases of the respiratory tract (*Legionella pneumophila*), and others considered opportunistic pathogens for man, or responsible for diseases that occur only in presence of specific characteristics predisposing such as in the elderly, the immunocompromized, in people with serious chronic illness or subjected to prolonged antibiotic treatment. Among these were tested the bacterium *Pseudomonas aeruginosa* and the fungi *Candida albicans*.

With respect to the mechanism of action of the Ag-loaded surface, it should be considered that Ag is long known for its antimicrobial effect and the antimicrobial property of Ag is being extensively researched with renewed interest in the recent past [23]. The exact mechanism by which Ag exert its killing effect on bacteria and viruses is still mainly obscure. However, it has been consistently observed that Ag and Ag ions can interact with microbes in different ways. For instance, silver can interact with the structural proteins on the surface of extracellular viruses to inhibit infection in the early phase, by either preventing viral attachment or entry, or by damaging the surface proteins to affect the structural integrity of virions [11, 12]. In the case of bacteria, it has been shown that the interaction between silver and the constituents of the bacterial membrane can cause structural changes and damage to the membranes and intracellular metabolic activity, which might be the cause or consequence of cell death [11].

## Concluding remarks and perspectives

Nowadays, the outbreak of COVID-19 brings biosafety to the forefront of public opinion, strengthening the necessity for related scientific research in the field of new materials for microorganism control. Notably, in the course of COVID-19 pandemic, many national governments have encouraged the development of new approaches, which puts forward higher requirements for researchers in biosafety and relevant fields [2].

The search of new self-disinfecting surfaces appears to be mandatory considering the serious consequences of the pandemic outbreaks leading to high costs of resolution, block in daily activities, the economic recession of regions and countries and in severe cases death.

Antibiotics, chemical disinfectants, and other forms of microbial biocides are highly effective against microorganisms. However, these effects are temporal as they wear off fairly quickly or diminish over a short period of time. Therefore, constant reapplication is required to keep up with disinfection [24]. This can be difficult, expensive, and sometimes logistically inefficient.

In this respect, the development of new surface treatments guarantying a long-term, intrinsic antimicrobial activity is of significant importance in reducing the risk of contamination in work and public places. In this respect, different approaches have been recently described in the scientific literature, including among them, the use of silver as a powerful antimicrobial and inhibitor of some of the mechanisms of viral infection [24], the specific value on the Coronavirus families and of SARS-CoV2 appears to be of particular interest [25].

Other surface treatments methods, specifically developed for the production of biomedical implants, rely on the nanotexturization, using electrochemical anodization that allows the formation of TiO2 nanotubes (TNTs), which substantially alter the physico-chemical properties of the material surface making it friendlier to human cells and promoting potential antibacterial properties [26]

Further approaches were reviewed by K. Bazaka and colleagues describing the strategies that confine antibacterial and/or antifouling property to the surface of the materials, by modifying the surface chemistry and morphology or by encapsulating the material in an antibiotic-loaded coating. Among them, they discussed the plasma-assisted modification of surface by nano structuring, chemical activation, and deposition of biologically active and passive coatings [27].

As herein described, GHA represents a material with intrinsic antimicrobial properties and elevated mechanical, tribological and anti-corrosive features. The GHA treatment with silver ions is indeed of significant importance in reducing the risk of contamination in the workplace, preventing epidemic outbreaks and containing the spread also with a view to safely resuming socialization activities in public places.

The electrochemical preparation of the golden hard anodizing GHA treatment was comprehensively described: the significant aspects to obtain protective coatings, related to the control of the process parameters, were detailed. The tribological characterization of the treatment was performed through unlubricated pin-on-disk wear tests. From the SEM/EDS analyses carried out on the worn surfaces, the main wear mechanisms detected were abrasion and tribo-oxidation. Specifically, at 250 m of sliding distance, the wear was characterized by the formation of surface oxide scales that reduce the contact between the two bodies and act as a sort of solid lubricant, reducing friction. Differently, at 20 m of sliding distance, a three-body abrasion, causing plastic deformation of the disk surface, took place.

The corrosion resistance of GHA was examined by neutral salt spray tests for different lengths of time: *t*=0 h, *t* = 500 h, and *t* = 1000 h. No significant differences were detectable after 500 h of treatment, whereas after 1000 h the surface presented general corrosion, with large, corroded areas. The obtained results revealed that the presence of Ag+ ions had a beneficial effect on the corrosion resistance of the GHA.

The antibacterial activity of GHA was analyzed by specific tests, described in the ISO 22196:2011 standard. The samples showed an appreciable bactericidal efficacy: *R* values ranged from 3.6 (in the case of Escherichia coli) to 4.2 (in the case of Staphylococcus aureus). Besides, the antibacterial activity remained constant for each tested thickness of treatment, indicating that the bactericidal activity of GHA was due to the treatment itself (i.e., the presence of Ag ions). Taken together, the specific features GHA indicate that such materials has a number of specific requisites in the development of specimens able to withstand intense daily usage and surface results in a “health surface” capable of contrasting the spread of the microorganisms, including viruses.

## References

[1] Sun D, Babar Shahzad M, Li M, Wang G, Xu D (2015). Antimicrobial materials with medical applications. Mater. Technol..

[2] Yu Y, Bu F, Zhou H, Wang Y, Cui J, Wang X, Nie G, Xiao H (2020). Biosafety materials: An emerging new research direction of materials science from the COVID-19 outbreak. Mater. Chem. Front..

[3] Walji SD, Aucoin MG (2020). A critical evaluation of current protocols for self-sterilizing surfaces designed to reduce viral nosocomial infections. Am. J. Infect. Control.

[4] Querido MM, Aguiar L, Neves P, Pereira CC, Teixeira JP (2019). Self-disinfecting surfaces and infection control. Colloids Surf. B: Biointerfaces.

[5] S. Rigo, C. Cai, G. Gunkel-Grabole, L. Maurizi, X. Zhang, J. Xu, C.G. Palivan, Nanoscience-Based Strategies to Engineer Antimicrobial Surfaces. Adv. Sci. **5**, 1700892 (2018) 10.1002/advs.201700892 PMC5979626 29876216

[6] Ateş S, Baran E, Yazıcı B (2018). The nanoporous anodic alumina oxide formed by two-step anodization. Thin Solid Films.

[7] Diggle JW, Downie TC, Goulding CW (1969). Anodic oxide films on aluminum. Chem. Rev..

[8] Georgantzia E, Gkantou M, Kamaris GS (2021). Aluminium alloys as structural material: A review of research. Eng. Struct..

[9] Kim HS, Kim DH, Lee W, Cho SJ, Hahn JH, Ahn HS (2010). Tribological properties of nanoporous anodic aluminum oxide film. Surf. Coat. Technol..

[10] Takahashi H, Chiba M (2018). Role of anodic oxide films in the corrosion of aluminum and its alloys. Corros. Rev..

[11] Woo KJ, Hye CK, Ki WK, Shin S, So HK, Yong HP (2008). Antibacterial activity and mechanism of action of the silver ion in Staphylococcus aureus and Escherichia coli. Appl. Environ. Microbiol..

[12] Gabe DR (2002). Hard anodizing - What do we mean by hard?. Met. Finish..

[13] T. Kinju, S. Rittoshi, T. Ikeda, S. Otsushi, Y. Matsuo, S. Koga-gun, EP 1207220A1 (2002)

[14] ISO 4287 - Geometrical Product Specifications (GPS) - Surface texture: Profile method - Terms, definitions and surface texture parameters, International Organization for Standardization (1997)

[15] ASTM International, ASTM G99-17, Standard Test Method for Wear Testing with a Pin-on-Disk Apparatus, Annual Book of ASTM Standards (2017)

[16] Li X, Sosa M, Olofsson U (2014). A pin-on-disc study of the tribology characteristics of sintered versus standard steel gear materials. Wear.

[17] E.N. Iso EN ISO 9227 (2017) [Online]. Available: http://store.uni.com/catalogo/uni-en-iso-9227-2017. Accessed Dec 2020

[18] G. Leitch, S. Verhoeven, ISO 22196 Plastics- Measurement of antibacterial activity on plastic surfaces, GAP EnviroMicrobialServices (2014)

[19] Gadelmawla ES, Koura MM, Maksoud TMA, Elewa IM, Soliman HH (2002). Roughness parameters. J. Mater. Process. Technol..

[20] Bolelli G, Cannillo V, Lusvarghi L, Manfredini T (2006). Wear behaviour of thermally sprayed ceramic oxide coatings. Wear.

[21] Kennedy FE, Lu Y, Baker I (2015). Contact temperatures and their influence on wear during pin-on-disk tribotesting. Tribol. Int..

[22] M. Varga, H. Rojacz, H. Winkelmann, H. Mayer, E. Badisch, Wear reducing effects and temperature dependence of tribolayer formation in harsh environment. Tribol. Int. **65**, 190–199 (2013)

[23] Galdiero S, Falanga A, Vitiello M, Cantisani M, Marra V, Galdiero M (2011). Silver nanoparticles as potential antiviral agents. Molecules.

[24] Schmidt MG, Fairey SE, Attaway HH (2019). In situ evaluation of a persistent disinfectant provides continuous decontamination within the clinical environment. Am. J. Infect. Control.

[25] Jeremiah SS, Miyakawa K, Morita T, Yamaoka Y, Ryo A (2020). Potent antiviral effect of silver nanoparticles on SARS-CoV-2. Biochem. Biophys. Res. Commun..

[26] Kunrath MF, Leal BF, Hubler R, de Oliveira SD, Teixeira ER (2019). Antibacterial potential associated with drug-delivery built TiO_2_ nanotubes in biomedical implants. AMB Express.

[27] Bazaka K, Jacob MV, Chrzanowskic W, Ostrikov K (2015). Anti-bacterial surfaces: natural agents, mechanisms of action, and plasma surface modification. RSC Adv..

